# Which Cognitive Factors Predict L2 Grammar Learning: Cognitive Control, Statistical Learning, Working Memory, or Attention?

**DOI:** 10.3389/fpsyg.2022.943988

**Published:** 2022-07-14

**Authors:** Yao Chen, Li Li, Mengxing Wang, Ruiming Wang

**Affiliations:** ^1^School of Foreign Studies, South China Normal University, Guangzhou, China; ^2^Key Laboratory of Chinese Learning and International Promotion, School of International Culture, South China Normal University, Guangzhou, China; ^3^Philosophy and Social Science Laboratory of Reading and Development in Children and Adolescents (South China Normal University), Ministry of Education, Guangzhou, China

**Keywords:** individual difference, cognitive control, working memory, statistical learning, attention, L2 grammar learning

## Abstract

Individual variability of cognitive factors in second language (L2) grammar learning has long been the focus in the field of L2 acquisition. Most explored the issue by focusing on one factor like cognitive control, working memory, statistical learning (SL), or attention. Few investigated the topic by taking all these factors into consideration. However, different factors might interact and collaboratively contribute to the learning process. Examining the issue by considering all the factors might yield different results and facilitate our understanding of the mechanism subserving L2 grammar learning. Therefore, this study explored whether and how these factors predicted L2 grammar learning. A total of 34 college students completed a set of cognitive measurements on these cognitive factors, after which they were trained with artificial grammar over 5 consecutive days. Using multiple regression analysis and machine learning algorithms, we found that in the initial phase, SL was the more significant predictor, whereas in the intermediate and the last phases, cognitive control served as the more significant predictor. In other words, in the initial phase of L2 grammar learning, SL might play an important role, whereas in the intermediate and proficient phase, the updating component of cognitive control might play a more significant role. The findings provided empirical evidence to the neurocognitive account of grammar learning, shedding light on the mechanism of L2 grammar learning.

## Introduction

Learning the grammatical patterns of a second language (L2) has been a struggle for most adults (Friederici, 2017; Luque and Morgan-Short, 2021). However, for some adults, it can be relatively effortless. Such individual variability in grammar learning has attracted a line of research to explore the cognitive factors that can account for such variances (Luque and Morgan-Short, 2021). Most of the extant studies mainly focused on the predictive power of one individual cognitive factor in L2 grammar learning like cognitive control, statistical learning (SL), working memory, or attention (e.g., Linck and Weiss, 2015; Stone and Pili-Moss, 2016; Sagarra, 2017; Zhou et al., 2017; Issa and Morgan-Short, 2019; Godfroid and Kim, 2021; Luque and Morgan-Short, 2021). However, most of these studies merely examined the predictive effect of an individual cognitive factor on L2 grammar learning, neglecting the cooperative effect of these factors on grammar learning. According to the neurocognitive account of grammar learning (Skeide et al., 2016; Friederici, 2017), cognitive factors would collaborate to subserve the process of grammar learning. To be specific, in the initial phase, the learning process is input-driven, with more involvement in the left temporal cortex and ventral language neural network; namely, to acquire the grammatical rules of a new language, individuals would rely on the statistical properties of the language input to extract the underlying grammatical patterns. As proficiency develops, learners would employ merge operation, namely, merging two constituents to form syntactic representation, to further process the grammatical patterns, while in the intermediate and more proficient phase, grammar learning would be a top–down process, with more involvement in the bilateral frontal cortex and dorsal language network. In other words, they might resort to a mechanism for complex syntactic pattern processing and other higher-order cognitive functions such as cognitive control (Chen et al., 2021a,b). This suggests that different cognitive factors might play different roles in different phases of L2 grammar learning, which might result in the different predictive power of these cognitive factors during the learning process.

Thus, the current study tended to comprehensively examine how different cognitive factors accounted for the individual variance in L2 grammar learning across different phases of the learning process, through which the underlying mechanism of L2 grammar learning could be examined.

### The Predictive Power of Cognitive Control on L2 Grammar Learning

There have been fruitful findings on the bilingual advantage in cognitive control (e.g., Bialystok et al., 2004, 2008, 2012; Martin-Rhee and Bialystok, 2008). This encouraged researchers to consider whether it was the bilingual experience that resulted in such an advantage, or it was the better competence in cognitive control that led to the preference of bilinguals in learning a new language. This has spurted numerous studies to explore the predictive power of cognitive control on L2 learning, among which only a few focused on L2 grammar learning (e.g., Linck and Weiss, 2015; Stone and Pili-Moss, 2016).

Linck and Weiss (2015) investigated whether cognitive control could predict L2 grammar attainment. They asked the participants to finish the cognitive control task (Simon Task) and the L2 grammar test after their short period of natural language learning. Linck and Weiss did not find any predictive power of cognitive control on L2 grammar attainment in the initial phase of learning. Similarly, Stone and Pili-Moss (2016) explored whether cognitive control could predict the performance of L2 grammar learning. They invited the participants to perform cognitive control task (Flanker task) and to learn the artificial language with complex grammatical patterns. Stone and Pili-Moss also did not find any predictive power of cognitive control on L2 grammar learning. However, these studies only examined the topic with one single cognitive control task and merely focused on the initial phase of grammar learning, which was not plausible in examining the issue since cognitive control was comprised of inhibition, switching, and updating components (Miyake et al., 2000), and grammar learning is a natural developmental process. This consequently resulted in the absence of the predictive power of cognitive control in L2 grammar learning.

The studies with multiple tasks on cognitive control and with a focus on the intermediate and proficient phases of grammar learning yielded divergent results. For example, Kapa and Colombo (2014) examined the issue with the digit span task for measuring the updating component, the Simon Task for the inhibition component, and the Wisconsin Card Sorting Task for the switching component. Besides, Kapa and Colombo trained the participants with artificial language, after which they found that the inhibition component could significantly predict the performance of L2 grammar learning. Taking the multi-facet nature of cognitive control into consideration, Luque and Morgan-Short (2021) examined the issue by asking the participants with intermediate proficiency in L2 to perform a set of complex tasks for cognitive control measurements, including the Flanker task for measuring the inhibition component and the Automated Continuous Performance Task for more complex measurement on the capacity to inhibit the irrelevant information during cognitive processing. Additionally, the participants’ proficiencies in natural language were also measured. Finally, Luque and Morgan-Short found the predictive power of cognitive control on L2 composite proficiency that incorporated the performance of grammar learning. Such consistencies in the findings indicate that the predictive power of cognitive control on L2 grammar learning can be found in the intermediate and proficient phases of the learning process. Besides, since young adults are at the peak of their cognitive functioning, resorting to multiple tasks or more complex measurements of cognitive control can render more opportunities to observe the role of cognitive control in L2 grammar learning (Luque and Morgan-Short, 2021). However, concerning the specific component within cognitive control that might play a crucial role in grammar learning, the answer remains vague. This is because, although the abovementioned studies seemingly found the inhibition component as the most important predictor in grammar learning, they did not directly examine the relationship between cognitive control and L2 grammar learning. Specifically, the grammar underlying the artificial language used in Kapa and Colombo’s study was merely simple linear order of the artificial vocabularies, which makes the results more representative of the lexical aspect of L2 learning (Kapa and Colombo, 2014). Besides, although the L2 composite score used in Luque and Morgan-Short’s study incorporated grammar learning performance, the outcome still gets influenced by other linguistic aspects in the measurement. Therefore, the direct examination concerning the role of the specific component within cognitive control in L2 grammar learning merits further investigation.

### The Predictive Power of Statistical Learning on L2 Grammar Learning

Statistical learning refers to a kind of cognitive mechanism responsible for extracting the statistical rules from the environmental input (Saffran et al., 1996), whose theoretical rationale is similar to the counterpart of language learning that also incorporates the extraction and integration of underlying rules from linguistic input (Kidd, 2012). This has attracted burgeoning research on the role of SL in language learning (e.g., Qi et al., 2019; van der Kleij et al., 2019). At first, these studies merely examined the issue by considering SL as a unified construct. However, as more and more studies found different developmental trajectories of SL mechanism across different modalities such as visual and auditory modalities, researchers in the field came to realize that SL might be a multicomponent mechanism that would get altered across different modalities and different domains such as verbal and non-verbal domains (e.g., Siegelman and Frost, 2015). Conway (2020) also pointed out that different modalities and different domains would alter the neural substrates of SL; for example, visual SL would activate the visual cortex like the occipital lobe while auditory SL would activate the auditory cortex like the temporal lobe. As driven by this account, many researchers employed SL tasks across different modalities while examining the role of SL in language learning. For example, Qi et al. (2019) explored the role of visual SL and auditory SL in reading, and they found that the role of auditory SL outperformed visual SL in reading. This further indicates the significance of examining the issue with modality into consideration (Qi et al., 2019).

Among all the extant studies, most of them focused on lexical learning and reading. Only a few studies examined the role of SL in grammar learning (e.g., Misyak and Christiansen, 2012; Kidd and Arciuli, 2016; Daltrozzo et al., 2017), among which only Godfroid and Kim (2021) focused on L2 grammar learning. Goldforid and Kim explored the role of SL in L2 grammar learning with SL tasks in different modalities such as the visual non-verbal task and the auditory non-verbal task. Besides, they asked the participants who were highly proficient in L2 to complete a set of measurements of their L2 grammatical attainment. However, Goldforid and Kim did not find a robust association between SL and L2 grammar learning with SL tasks, let alone the predictive power of SL on L2 grammar learning. This might be because, first, they neglected the modulatory effect of domain features in the SL mechanism, hindering us from observing the contribution of SL to L2 grammar learning. Second, they only examined the issue with highly proficient L2 learners in focus. However, according to the neurocognitive account of grammar learning, extraction and integration of statistical rules might only play a significant role in the initial phase of grammar learning rather than in the intermediate and proficient phases. Therefore, it is reasonable that Goldforid and Kim failed to observe SL as a predictor in the proficient phase of grammar learning. Examining the issue in the initial phase of the learning process might yield divergent results. More studies are needed to investigate the predictive power of SL in L2 grammar learning with considering the multicomponent nature of the SL mechanism and the developmental nature of L2 grammar learning. Moreover, concerning how SL predicted L2 grammar learning compared with other cognitive factors, the answer remains vague. By examining the issue with other cognitive factors, we can observe how SL contributes to grammar learning relative to other factors (Xu et al., 2020).

### The Predictive Power of Other Cognitive Factors on L2 Grammar Learning

In addition to cognitive control and SL, the predictive power of working memory on L2 grammar learning has also been the focus. Working memory is responsible for the maintenance and activation of short-lived memory during cognitive processing, which incorporates information storage and processing (Sagarra, 2017). Since L2 grammar learning necessitates the maintenance of the grammatical patterns extracted from linguistic input, working memory might participate in L2 grammar learning (Zhou et al., 2016, 2017).

To examine the issue, Brooks and Kempe (2013) used the non-word repetition task as a working memory measurement. However, this task only taps into the information storage of working memory while grammar learning necessitates not only information storage but also processing, leading to the absence of the predictive power of working memory. To address this issue, some studies used complex span measurements such as the operation span task for exploration (e.g., Zhou et al., 2016, 2017). For example, Zhou et al. (2016, 2017) examined the role of working memory in the proficient phase of syntax processing and syntax production with operation span task as measurement. Zhou et al. (2016, 2017) found that working memory could serve as a predictor of L2 syntax processing, suggesting a role of working memory in L2 grammar learning. However, concerning whether the role will get modulated when other cognitive factors are examined, there is still much space for investigation.

In addition, according to the noticing hypothesis, learners needed to pay attention to the linguistic patterns before they acquired a new language (Schmidt, 1990). This was supported by the empirical evidence which found that with attention, the novel linguistic patterns could be learned better (e.g., Tanaka et al., 2008). This suggests that attention might play a role in L2 development, which has drawn numerous studies to examine the account (e.g., Godfroid et al., 2013; Loewen and Inceoglu, 2016). However, the relevant studies on L2 grammar learning are scarce. Issa and Morgan-Short (2019) explored the role of attention in L2 grammar learning by modulating the input features to alter participants’ level of attention, and they found that a higher level of attention led to better syntax processing. Notwithstanding, the study explored the issue without tapping into the cognitive mechanism of attention (Issa and Morgan-Short, 2019). Attention was comprised of alerting, orienting, and executive control (Fan et al., 2002), each of which might exert a different influence on L2 grammar learning. Exploring the role of these components within the attention mechanism in L2 grammar learning might offer us a more comprehensive understanding of the issue.

### The Predictive Power of Combined Cognitive Factors on L2 Grammar Learning

Most of the abovementioned studies just separately examined the predictive power of individual cognitive factors on L2 grammar learning. Seldom have any studies examined the issue by taking into consideration all these factors. However, as pointed out by the neurocognitive account of grammar learning, different factors may contribute to L2 grammar learning collaboratively in different phases of the learning process (Kormos, 2012; Skeide et al., 2016; Friederici, 2017). Thus, exploring the issue by considering more than one cognitive factor might, for one thing, render divergent findings from the studies which merely examined a single cognitive factor (e.g., Linck and Weiss, 2015). For another, it might also unveil the underlying mechanism of L2 grammar learning, lending support, and refinement to the neurocognitive account of grammar learning.

For example, Linck and Weiss (2015) examined the combined roles of cognitive control and working memory in the initial phase of L2 grammar learning. Specifically, they asked the participants to finish the operation span task for working memory measurement and the Simon Task for cognitive control measurement. Additionally, they also required participants to report their average grade for language competence measurement. Linck and Weiss found that in the initial phase of learning, only working memory was related to L2 grammar learning, whereas they failed to obtain any association between cognitive control and L2 grammar learning. The results indicate a more significant role of working memory rather than cognitive control in the initial phase of L2 grammar learning, further suggesting that cognitive control might not play a crucial role at the beginning of the learning process. However, since Linck and Weiss only focused on two cognitive factors in the study, they could not investigate whether working memory still plays a significant role in the initial phase of learning when other factors are considered, especially SL since it was assumed by the neurocognitive account of grammar learning to play a crucial role in the initial phase of learning. Whether working memory interacts with SL to subserve the initial phase of grammar learning? or whether the role of working memory would be masked by SL in this phase of learning? All these questions merit further investigation. In addition, the study only focused on the initial phase of learning, obscuring the results from revealing the role of cognitive control in the intermediate and proficient phase of grammar learning. Therefore, more studies with longitudinal design are needed to explore the predictive power of multiple cognitive factors in the developmental process of L2 grammar learning, whose results might, to a degree, directly examine the neurocognitive account of grammar learning.

## The Current Study

In recognition of the abovementioned gaps, the current study tended to explore the predictive power of the abovementioned cognitive factors in the development of L2 grammar learning, including cognitive control, SL, working memory, and attention. The research questions were whether and how these cognitive factors predicted L2 grammar learning? Addressing the questions can, first, illuminate which cognitive factors account for the individual variance in L2 grammar learning and, second, facilitate our understanding of the underlying cognitive mechanism subserving L2 grammar learning. Abiding by the neurocognitive account of grammar learning (Skeide et al., 2016; Friederici, 2017), we hypothesized that SL might serve as the most significant predictor of L2 grammar learning in the initial phase of learning since statistical regularity extraction was assumed to be important in this phase, and cognitive control might act as the most important predictor in the intermediate and proficient phases of the learning process.

## Materials and Methods

To answer the research questions, we used a set of measurements on the cognitive factors. Besides, we employed a longitudinal design of the artificial grammar learning paradigm to observe the developmental process of L2 grammar learning. The artificial grammar learning paradigm was used because, first, artificial grammar can rid the results from the influence of confounding factors in a language like semantic factors (Petersson et al., 2012). Second, many studies found that the neural mechanisms of artificial grammar learning partially overlapped with the counterpart of L2 grammar learning (Petersson et al., 2012). All of these justified the artificial grammar learning paradigm as a proper window for observing L2 grammar learning.

### Participants

A total of 34 college students participated in the study, all of whom were Chinese native speakers with ages around 22 and 27 (*M* = 23.74; *SD* = 1.31). They all had normal vision and hearing and they had no reported language disorders. Before the experiment, they finished Language History Questionnaire 3.0 (Li et al., 2020), whose results indicated that the participants have acquired Chinese since birth and began learning English as their L2 at the age of around 10. None of them had the experience of studying abroad or living abroad. Their average proficiency in Chinese was significantly higher than in English (*M* Chinese = 0.717, *SD* = 0.159; *M* English = 0.656, *SD* = 0.117; *t* = 1.945, *df* = 32, *p* = 0.030). They all provided written informed consent and received monetary compensation for their participation in the experiment.

### Research Methods

The current study employed the tasks in measuring the competence of cognitive control, working memory, SL, and attention. Besides, an artificial grammar learning paradigm was also used in the experiment.

#### Cognitive Control Tasks

Cognitive control was comprised of inhibition, shifting, and updating (Miyake et al., 2000). These components could be measured with the Flanker task, more odd shifting task, and N-back task (Miyake et al., 2000; Friedman and Miyake, 2017; Zhang et al., 2018).

In the Flanker task, participants were presented with a sequence of arrows, and they were asked to judge the direction of the central arrow that was accompanied by the other arrows within the sequence. The directions of the other arrows were congruent or incongruent with the directions of the central arrows (Luk et al., 2010). In the more odd shifting task, participants were presented with a number at the center of the screen one at a time, and they needed to judge whether the number was larger than 5 or an odd or even number according to the color signal (Zhang et al., 2018). To be specific, if the number was displayed in red, the participants shall judge whether the number was larger than 5. If the number was in green, the participants shall judge whether the number was an odd or even number. The N-back task incorporated 0-back, 1-back, and two-back tasks. The 0-back task mainly required the participants to judge whether the letter presented on the screen was identical to the pre-specified letter. Then, The 1-back and 2-back tasks required the participants to judge whether the presented letter was identical to the one or two items before its onset (Miyake et al., 2000; Yang and Li, 2012).

#### Statistical Learning Tasks

The paradigm used for SL measurement was the classic SL task (Saffran et al., 1996). Given that SL would get altered across different modalities and different domains, the SL tasks we used included the auditory-verbal task, the auditory non-verbal task, the visual verbal task, and the visual non-verbal task (Schneider et al., 2020). The difference among these tasks lies in the experimental stimulus. For the auditory-verbal task, 12 English syllables were selected as stimuli, and they were pi, pu, pa, ti, tu, ta, di, du, da, bi, bu, and ba. These syllables were grouped into four target triplets, pa-bi-ku, go-la-tu, da-ro-pi, and ti-bu-do. For the auditory non-verbal task, 12 musical tones within the same octave were selected as stimuli, and they were F, G, D, G#, C#, B, C, F#, D#, E, A, and A#. These tones were grouped into four target triplets, F#DE, ABC, C#A#F, and GD#G#. For the visual verbal task, 12 letters were chosen as stimuli, including B, J, K, A, H, C, F, E, J, G, D, and M. These letters were organized into four target triplets, GJA, FKC, LBE, and MDH. For the visual non-verbal task, 12 alien cartoon images were included as stimuli which were also structured into four target triplets. For each task, the target triplets got repeated 24 times in visual tasks and 48 times in auditory tasks. These target triplets were concatenated into continuous streams for each task.

Despite the difference in stimulus, the experimental procedures were identical across the SL tasks. For each task, there was a familiarization phase and a test phase. In the familiarization phase, the participants were exposed to a continuous stream of triplets. In the test phase, the participants had to finish a two-alternative forced-choice task where they would be presented with two triplets, one occurred in the familiarization phase whereas the other one did not, and they shall determine which triplet they were more familiar with.

#### Working Memory Tasks

Working memory could be observed with the complex span task such as the operation span task during which participants were first presented with a stream of letters (Unsworth et al., 2005; Zhou et al., 2016, 2017). Subsequently, the participants would be asked to perform math problems, after which they were presented with a list of letters. Then, they had to pick up the letter presented before among the list of letters. The accuracy of math problems shall be above 85%, or else the data would be ruled out from the analysis.

#### Attention Task

Attention was comprised of alerting, orienting, and executive control, which could be measured with the attention network test (Fan et al., 2002). The test required the participants to determine the direction of the central arrows on the screen. The central arrows would be flanked by an array of arrows whose directions were incongruent or congruent with the central arrows. Besides, no cues, center cues, and spatial cues would be presented before the occurrence of the arrows, among which no cues provided no information, center cues, and double cues also provided no information but could alert participants’ attention, and spatial cues could provide predictive spatial information of the imminent appearance of the arrows. Alerting component was measured by the changes in reaction time between the trials with no cues and the trials with double cues; orienting was examined by the difference in reaction time between the trials with center cues and those with spatial cues; executive control was measured with the reaction time difference between the trials with congruent flankers and those with incongruent flankers (Fan et al., 2002).

#### Artificial Grammar Learning Paradigm

The artificial grammar learning (AGL) paradigm was from Yang and Li (2012), which involved grammatical sequences that were generated through finite-state grammar. Finite-state grammar refers to a complex system that can derive the grammatical sequences *via* connecting the nodes abiding by the paths pre-specified in the grammatical circuit, e.g., pok kun pok (Reber, 1967; Petersson et al., 2012; Silva et al., 2017), while ungrammatical sequences are generated by switching one or two nodes of the grammatical sequences, which did not follow the paths in the grammatical circuit, e.g., pok kun (Silva et al., 2017). Besides, the sequences were alphabets that shared more similarities with alphabetic languages such as English. Thus, these sequences were pronounceable. The AGL paradigm incorporated the exposure phase and the test phase. In the exposure phase, participants were exposed to the visual grammatical sequences, after which participants needed to memorize the sequence by typing it on a blank screen. In the test phase, participants were exposed visually to the grammatical sequences and the ungrammatical sequences. During this process, participants needed to finish a grammatical judgment task where they had to discriminate the grammatical sequences from the ungrammatical sequences. Moreover, since we wanted to explore the developmental process of L2 grammar learning, we trained the participants with the artificial grammar sequences over 5 consecutive days. The accuracy rate of the grammatical judgment task severed as the performance of grammar learning.

## Results

To observe the predictive power of the abovementioned cognitive factors on L2 grammar learning, we used multiple regression analysis. To further examine the results, we complemented machine learning algorithms. This is because traditional regression analysis only inferences the result patterns from a given dataset while machine learning can not only extract the patterns from a given dataset but also predict the patterns in a new dataset. In other words, the traditional approach addresses the problem concerning whether the independent variables predict the dependent variables, while machine learning can address the question of how well the independent variables predict the dependent variables (Rosenbusch et al., 2021). Therefore, this renders more reliability and generalizability of the results with machine learning analysis, making the results more replicable and generalizable (Orrù et al., 2020). Besides, since grammar training lasted for 5 days, we conducted the analyses separately for each day of the training. Multiple regression analyses were conducted with R (R Core Team, 2021), and machine learning analyses were conducted *via* Python (van Rossum and Drake, 2021).

For day 1 training, we constructed the maximal model with all the measured cognitive factors as independent variables and with the performance of the grammatical judgment task of day 1 training as the dependent variable. Then, we used the backward approach to determine the best fitting model, after which we obtained the results of the best fitting model. The results showed that 44.49% of the variance in the performance of grammar learning could be explained by the predictors of the best-fitting model collectively, *F*(9,24), *p* = 0.003. The results also revealed that grammar learning performance on day 1 could be predicted by the performance of the visual non-verbal SL task (β = 0.283, *t* = 3.745, *p* = 0.001), the auditory non-verbal SL task (β = 0.269, *t* = 2.919, *p* = 0.008), and the orienting effect of attention network test (β = 0.03, *t* = 2.918, *p* = 0.008). Besides, we checked the assumptions of homogeneity of variance, linearity, and normality of residuals, and we found that the best-fitting model did not violate these assumptions.

For day 2 training, we constructed the maximal model and obtained the best-fitting model in the identical way as we did in the day 1 analysis. The results showed that 33.88% of the variance in the performance of grammar learning could be explained by the predictors of the best-fitting model, *F*(7,26), *p* = 0.010. The results also revealed that the grammar learning performance on day 2 could be predicted by the performance of the N-back task (β = 0.452, *t* = 2.765, *p* = 0.010). The best-fitting model did not violate the assumptions of multiple regression analysis.

For day 3 training, we constructed the maximal model and obtained the best-fitting model. The results showed that 60.62% of the variance in the performance of grammar learning could be explained by the predictors of the best-fitting model, *F*(9,24), *p* < 0.001. The results revealed that the grammar learning performance on day 3 could be predicted by the performance of auditory verbal SL task (β = 2.379^*e–*01^, *t* = 3.244, *p* = 0.003), auditory non-verbal SL task (β = 2.395^*e–*01^, *t* = 3.501, *p* = 0.001), N-back task (β = 6.699^*e–*01^, *t* = 4.528, *p* = 0.000), the mixing cost of more odd shifting task (β = –3.178^*e–*04^, *t* = –3.392, *p* = 0.002), and the operation span task (β = –2.114^*e–*03^, *t* = –2.397, *p* = 0.025). From the analysis, auditory SL and updating component of cognitive control were positive predictors, while the shifting component of cognitive control and working memory competencies served as the negative predictors. The best-fitting model did not violate the assumptions of multiple regression analysis.

For day 4 training, we obtained the best-fitting model. The results showed that 33.32% of the variance in the performance of learning could be explained by the predictors of the best-fitting model, *F*(4,29), *p* = 0.003. The results revealed that the grammar learning performance on day 4 could be predicted by the performance of the auditory non-verbal SL task (β = 0.234, *t* = 2.234, *p* = 0.033), the N-back task (β = 0.610, *t* = 2.810, *p* = 0.009) and the mixing cost of more odd shifting task (β = –0.0003, *t* = –2.478, *p* = 0.019). Among the predictors, auditory non-verbal SL and updating component of cognitive control served as the positive predictors while the shifting component served as the negative predictor. The model did not violate the assumptions of multiple regression analysis.

For day 5 training, we obtained the best-fitting model in the same way. The results showed that 52.07% of the variance in the performance of grammar learning could be explained by the predictors of the best-fitting model, *F*(5,28), *p* < 0.001. The results revealed that the grammar learning performance on day 5 could be predicted by the performance of the auditory non-verbal SL task (β = 2.057^*e–*01^ *t* = 2.819, *p* = 0.009), the N-back task (β = 6.682^*e–*01^, *t* = 4.339, *p* = 0.000), and the mixing cost of more odd shifting task (β = –3.025^*e–*04^, *t* = –3.448, *p* = 0.002). Among these predictors, auditory non-verbal SL and updating component of cognitive control were the positive predictors while the shifting component of cognitive control was the negative predictor. The model did not violate the assumptions of multiple regression analysis.

In addition, for all the analyses, to observe whether the data met the assumption of collinearity, the tolerance values and variance inflation factor values (VIF) were calculated, which indicated that collinearity was not a concern in these analyses (visual-verbal SL, tolerance = 0.644, VIF = 1.553; visual non-verbal SL, tolerance = 0.660, VIF = 1.516; auditory-verbal SL, tolerance = 0.699, VIF = 1.431; auditory non-verbal SL, tolerance = 0.721, VIF = 1.388; Flanker effect, tolerance = 0.728, VIF = 1.373; N-back, tolerance = 0.647, VIF = 1.545; alerting effect of attention network test, tolerance = 0.641, VIF = 1.560; orienting effect of attention network test, tolerance = 0.527, VIF = 1.896; executive control of attention network test, tolerance = 0.590, VIF = 1.696; mixing cost of more odd shifting task, tolerance = 0.674, VIF = 1.484; switching cost of more odd shifting task, tolerance = 0.650, VIF = 1.539; working memory, tolerance = 0.550, VIF = 1.817).

**FIGURE 1:**
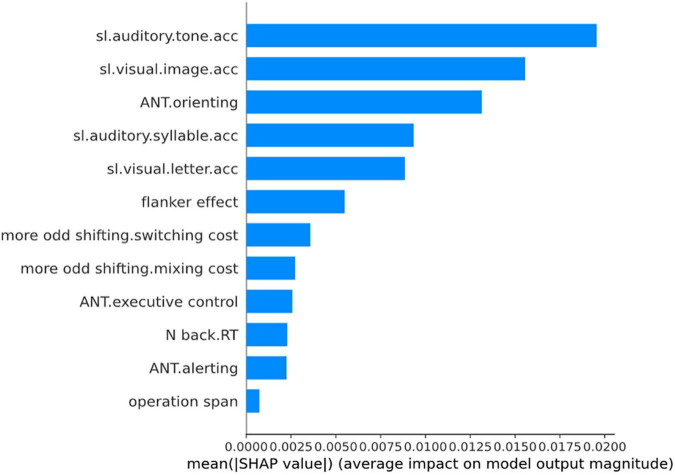
The feature importance plot for day 1 analysis. sl.aditory.tone.acc, sl.visual.image.acc, sl.auditory.syllable.acc, and sl.visual.letter.acc referred to SL task performance. ANT.orienting, ANT.executive function, and ANT.alerting referred to the orienting, executive function, and alerting components of attention, respectively. More odd shifting.switching cost and more odd shifting.mixing cost referred to the switching cost and mixing cost of more odd shifting task, respectively. Besides, the feature importance was ranked with mean SHAP values which were displayed on the *y*-axis in the figure.

To further examine the results, we used machine learning algorithms. The algorithms used in the study included regression methods and tree-based methods because we intended to observe the importance of the cognitive factors in L2 grammar learning. Regression methods included linear regression, lasso regression, ridge regression, and elastic net regression. The tree-based methods included one of the bagging algorithms such as random forest and one of the boosting algorithms such as XGboost. We first constructed the base models with the default hyperparameters of the algorithms, then we tuned the hyperparameters with the grid search method (LaValle et al., 2004). We subsequently constructed the best models with the best parameters obtained *via* the grid search method and compared the *r*^2^ values of these models to identify the best model (Rosenbusch et al., 2021). However, owing to the small sample size of the study, even with the best models constructed, we still obtained a negative *r*^2^ value for day 2 to day 4 analyses. Thus, we only reported the analyses on day 1 training. The best model was constructed with the random forest, and the *r*^2^ value was 0.188. We obtained the feature importance of the model with the Shapley additive explanations (SHAP) approach (Lundberg and Lee, 2017), and we obtained the ranking plot as illustrated in Figure 1.

Taken together, based on the β values of the significant predictors in the best-fitting models of multiple regression analyses for day 1 to day 5 training and the results of machine learning analysis for day 1 training, we summarized the numerical order of the predictive values of the cognitive factors in Table 1. From Table 1, we could know that in the initial phase (day 1), visual, auditory non-verbal SL, and the orienting component of attention could significantly predict the performance of L2 grammar learning. In the intermediate phase (day 2–day 3), the updating and shifting components of cognitive control, and auditory non-verbal SL could predict the performance of grammar learning. In addition, in the last phase (day 4–day 5), updating and shifting components of cognitive control and auditory non-verbal SL could serve as predictors of the performance of L2 grammar learning. Generally speaking, the findings suggest that in the initial phase of L2 grammar learning, SL serves as the more powerful predictor in L2 grammar learning, while in the intermediate and the last phases, cognitive control serves as the more significant predictor. This indicates that compared with the other cognitive factors, SL and cognitive control might play more influential roles in the development of L2 grammar learning.

**TABLE 1:** The predictive power of the cognitive factors on L2 grammar learning.

	The numerical order of the predictive power of cognitive factors
Day 1	Visual non-verbal SL and auditory non-verbal SL > attention-orienting
Day 2	Cognitive control-updating
Day 3	Cognitive control-updating > cognitive control-shifting (negative) > auditory non-verbal SL > auditory verbal SL > working memory (negative)
Day 4	Cognitive control-updating > auditory non-verbal SL > cognitive control-shifting (negative)
Day 5	Cognitive control-updating > auditory non-verbal SL > cognitive control-shifting (negative)

*Attention-orienting referred to the orienting component of attention; cognitive control-updating referred to the updating component of cognitive control; cognitive control-shifting (negative) referred to the shifting component of cognitive control, and this was a negative predictor; working memory (negative) signified that working memory was a negative predictor.*

## Discussion

The current study mainly examined the predictive power of cognitive factors on L2 grammar learning, including cognitive control, SL, working memory, and attention. Using multiple regression analysis and machine learning algorithms, we found that SL served as the significant predictor in the initial phase of grammar learning while cognitive control served as the important predictor in the intermediate and proficient phases. In other words, compared with the other cognitive factors, SL and cognitive control might play more important roles during the learning process. The findings were consistent with our hypotheses, which were discussed according to the different phases of the learning process as follows.

In the initial phase, visual, auditory non-verbal SL, and the orienting component of attention were found to significantly predict the performance of L2 grammar learning. In other words, in this phase, these factors might play important roles. This was inconsistent with the previous work (Godfroid and Kim, 2021), which could not robustly find a role of SL in L2 grammar learning. This might be because first, we considered the domain feature in the current study which rendered us more opportunities to observe the role of SL in L2 grammar learning. Besides, Gold and Kim only examined the issue with a focus on the proficient phase of grammar learning. However, we adopted a longitudinal design that made us more possible to observe the role of SL throughout the learning process. Thus, we obtained the involvement of SL in the initial phase of L2 grammar learning. This shed light on the neurocognitive account of grammar learning, which assumed that at the beginning, since the participants were unfamiliar with the artificial grammar sequences, they would tend to rely on the statistical properties from the linguistic input to acquire the grammatical patterns (Skeide et al., 2016; Friederici, 2017; Ullman, 2020). In addition, both visual SL and auditory SL were found to be crucial in this phase, which might be because the grammatical sequences were presented visually. Thus, it is understandable that participants employed the visual SL mechanism to extract the underlying rules of the visual sequences. Additionally, since the artificial sequences were pronounceable with the cues of visual graphemes, e.g., pok kun pok, participants might complement the use of the auditory cues to facilitate the extraction of the grammatical rules since phonological processing was found to play an important role in sequence reading (Qi et al., 2019).

Besides, that attention was found to play a role in the initial phase of learning which was in line with previous work that emphasized a significant role of attention in L2 grammar learning (Schmidt, 1990; Issa and Morgan-Short, 2019). This testified to the noticing hypothesis which assumed that a higher level of attention could lead to better L2 development (Schmidt, 1990). Additionally, the findings extended the understanding of the role of attention in L2 grammar learning from a unified construct to specific components of attention. Previous work only examined the issue by regarding attention as a unified construct (Issa and Morgan-Short, 2019). But in fact, attention was a multi-facet construct incorporating altering, orienting, and executive control (Fan et al., 2002), all of which were directly examined in this study. We found that it was the orienting component that could account for the individual variance of L2 grammar learning. This is a further step in observing the contribution of attention to L2 grammar learning. Moreover, attention was only found to play a role in the initial phase of learning. This might be because, in the initial phase, participants are completely unfamiliar with the sequences, so they needed to orient their attention to the linguistic input to extract the underlying grammatical patterns from the input. Nevertheless as proficiency increased, the process became more automatic where participants did not necessitate as much attention as they did in the initial phase.

In the intermediate phase, updating and shifting components of cognitive control and auditory SL were found to significantly predict the performance of L2 grammar learning. This suggests that in this phase, cognitive control and auditory SL played significant roles in L2 grammar learning.

Cognitive control was found to be a more influential predictor in this phase, which was consistent with the previous work that found the role of cognitive control in the intermediate phase of L2 grammar learning (Kapa and Colombo, 2014; Luque and Morgan-Short, 2021). The findings extended the understanding of this issue from whether cognitive control accounted for the individual variability in L2 grammar learning to which components of cognitive control explained the individual variance in the learning process. Contrary to the findings of prior work which found the inhibition component as the most important predictor (Kapa and Colombo, 2014; Luque and Morgan-Short, 2021), it was the updating and shifting components of cognitive control that played important roles in the learning process. The inconsistency might be because, as mentioned above in the Introduction section, the prior work did not directly explore the role of cognitive control in L2 grammar learning, confounding their results from the influence of other linguistic aspects in the language measurements they used. Nevertheless, this study directly examined the issue with grammar learning competence as the focus, leading to divergent results from the prior work. As for the involvement of updating in L2 grammar learning, it might be due to in this phase, after the extraction of grammatical patterns in the initial phase, participants had to hold the extracted rules in mind and maintain the representation of the current items. In this way, they could utilize the extracted rules to decode and process the current items. Since updating is responsible for holding the current information in mind to inhibit the interference of irrelevant information (Diamond, 2013), participants’ higher competence in updating led to better attainment in L2 grammar learning. In addition, even though the shifting component was found to predict L2 grammar learning performance, it was involved in the process as a negative contributor. In other words, higher competence in shifting would lead to worse learning performance. This might be because shifting is responsible for flexibly shifting from a set of previous knowledge to a new set of knowledge (Diamond, 2013). However, in the intermediate phase, the participants needed to stick to the extracted grammatical rules rather than shift between the newly learned rules and previous knowledge. Those with higher shifting capacity would be more likely to shift between newly learned grammatical rules and a set of previously learned rules of their native language, resulting in the fact that better shifting ability led to worse grammar learning performance. All in all, the findings testified to the neurocognitive account of L2 grammar learning that assumed the involvement of cognitive control in the intermediate phase of the learning process.

Moreover, that auditory SL served as a predictor of L2 grammar learning in the intermediate phase further verified the role of SL in L2 grammar learning. This might be because, in the intermediate phase, participants still needed to extract the grammatical patterns from the input to consolidate the memory of the extracted rules in the initial phase. This might also be because, in this phase, participants commenced using the merge operation, namely, merging two constituents to form syntactic representation, to process the linguistic sequences. The extraction and integration of linguistic rules underlying the merge operation resembled the function of SL, resulting in the role of SL in the intermediate phase of the learning process. However, it is interesting to find a role of auditory SL rather than visual SL in this phase of visual grammatical sequence learning. This might be because the grammatical sequences used in the current study were artificial. According to Gabay and Holt (2015) and Qi et al. (2019), phonological processing might play a more pivotal role in non-word sequence reading than in real-word sequence reading because non-word was more strongly associated with regularities detection in auditory input rather than visual input. Thus, in the current study, we observed a persistent role of auditory SL throughout the learning process rather than visual SL. Future studies might observe different patterns from ours if they used natural language sequences as experimental materials or materials which are not pronounceable.

Furthermore, working memory was also found to predict the performance of L2 grammar learning in this phase as a negative predictor. In other words, individuals with higher working memory competence displayed lower proficiency in grammar learning. The results concerning the role of working memory in the current study were not in line with prior work (Linck and Weiss, 2015; Zhou et al., 2016, 2017). To be specific, contrary to Linck and Weiss (2015) who found the positive role of work memory in the initial phase of grammar learning, we failed to observe the role of working memory at the beginning of the current study. This might be because, in the initial phase, participants did necessitate holding the sequences in mind with which SL mechanism could use to decode the underlying regularities. But unfortunately, since participants depended more on the SL mechanism in this phase (Skeide et al., 2016; Friederici, 2017), the role of working memory gets masked by the SL mechanism. This led to our failure in obtaining the role of working memory in the initial phase of grammar learning. However, in the intermediate phase, we found a negative role of working memory in grammar learning. This might be because after the initial phase of grammar learning, partial regularities were extracted and partial grammatical representation had been formed. All the participants had to do is to employ the updating component of cognitive control to hold the extracted regularities in mind. Then, these rules could serve the SL mechanism to further decode the upcoming sequences. However, higher working memory capacity in this phase might make the individuals more likely to maintain the memory of the surface structure of the upcoming artificial sequences, which would probably interfere with the grammatical rules extracted in the initial phase and influence the retrieval of the formed grammatical rules, leading to the worse performance in grammatical patterns learning. In addition, the inconsistencies between the findings of the current study and the previous work (Zhou et al., 2016, 2017) might lie in the language materials in use. Zhou et al. (2016, 2017) focused on natural language learning, while we used artificial grammar sequences for exploration. Despite many merits of using artificial grammar to research the process of grammar learning, artificial grammar sequences were devoid of semantics, complex phonological structures, and complex syntactic structures which are the features of natural language. Therefore, the information entropy of artificial grammar sequences was relatively lower than the counterparts of natural language sequences. With similar items of specific grammatical patterns in the artificial language, one might find it hard to decode and extract the underlying rules, henceforth hindering the process of grammar learning (Gómez, 2002). On the contrary, the complexity of semantics and phonological structures, and the flexibility of the surface structure of the natural language sequences would increase the information entropy. All this information could serve as the cues for learners to better decode the underlying regularities. Therefore, in the current study, those with higher working memory would tend to hold the artificial sequences with low information entropy in mind, which makes them get trapped in the surface structures of the artificial sequences, henceforth hindering them from further decoding the grammatical rules. However, in the study of Zhou et al. (2016, 2017), the participants with higher working memory would also tend to hold the natural language sequences in mind, but the rich linguistic information and flexible surface structure of the natural sequences would help with their regularity extraction and could facilitate their sequence processing. Consequently, this led to the different findings between the current study and prior work. More studies are needed to further unveil the role of working memory and its relationship with other cognitive factors during the grammar learning process.

In the last phase of the short-period training, updating and shifting components of cognitive control and auditory non-verbal SL were found to significantly predict the performance of L2 grmamar learning. Consistent with the previous work which found a role of cognitive control in the intermediate and proficient phase of learning (e.g., Luque and Morgan-Short, 2021), cognitive control was also found to play a role in the proficient phase in the current study. Similar to the intermediate phase, the positive involvement of updating component and the negative participation of the shifting component of cognitive control suggest that in this phase, to further decode and process the upcoming linguistic sequences, participants needed to maintain the extracted grammatical rules in mind rather than to shift between different sets of grammatical rules. The results further provided support to the neurocognitive account of grammar learning which found a role of cognitive control in the intermediate and proficient phases. Moreover, auditory SL was found to play a role in this phase of learning, which is inconsistent with the previous work which did not find a role of SL in the proficient phase of grammar learning (Godfroid and Kim, 2021). The inconsistency might be due to the difference in the language materials in use. To be specific, we used artificial sequences while Goldfroid and Kim measured the natural language grammar learning competence. As mentioned above, phonological processing plays a more pivotal role in non-word sequence processing than in real word processing (Qi et al., 2019), leading to the fact that the role of auditory SL in the proficient phase of grammar learning was found in the current study whereas no role of SL was found in this phase in prior work. This heeded us to take caution while drawing a conclusion with the findings. The role of SL in the proficient phase of grammar learning still necessitates more studies focusing on natural language learning for further investigation.

Taken together, SL was found to be the more significant predictor in the initial phase of L2 grammar learning, and the updating component of cognitive control was the more significant predictor in the intermediate and proficient phase of the learning process. This suggests that different cognitive factors might account for the individual variability in different phases of grammar learning, testifying to the plausibility of examining the issue with different factors and different phases of grammar learning into consideration. The findings also lend support to the neurocognitive account of grammar learning (Skeide et al., 2016; Chen et al., 2021a,b). According to the account, in the initial phase of grammar learning, learners principally rely on the statistical properties of the linguistic sequences to extract the grammatical patterns, resulting in the involvement of SL in the initial phase. As proficiency increases, learners commence merging two constituents to represent the grammatical patterns in mind, which also necessitates SL to extract and integrate the grammatical rules in the intermediate phase. When learners’ proficiencies get developed, they tend to employ cognitive control, especially the updating component, to maintain the extracted rules in mind to further process the upcoming sequences, leading to a critical role of cognitive control in the proficient phase of the learning process (Chen et al., 2021a,b).

## Conclusion

The current study aimed to comprehensively examine the predictive power of cognitive factors on L2 grammar learning including cognitive control, SL, working memory, and attention. It was found that in the initial phase, SL served as a more significant predictor, whereas in the intermediate and last phases, the updating component of cognitive control served as the more important predictor. The findings not only testified to the plausibility of examining the issue with multiple factors into consideration but also testified to the neurocognitive account of grammar learning. The results might, to a degree, facilitate our understanding of the underlying mechanism of L2 grammar learning. Moreover, the findings provide some implications for L2 grammar learning. Since SL might play a significant role in the initial phase, relevant training can be devised to improve learners’ SL competence before the learners commence learning the grammatical rules of L2. As proficiency increases, cognitive control training can be devised to facilitate the process of grammar learning. However, the sample size in the current study was relatively small, which rendered negative *r*^2^ values in the machine learning analyses. Future studies can recruit more participants to examine the issue and observe whether the findings can be validated. Moreover, future studies can base on natural language to further explore the issue, improving the ecological validity of the findings.

## Data Availability Statement

The raw data supporting the conclusions of this article will be made available by the authors, without undue reservation.

## Ethics Statement

The studies involving human participants were reviewed and approved by Human Research Ethics Committee for Non-Clinical Faculties; The School of Psychology, South China Normal University. The patients/participants provided their written informed consent to participate in this study.

## Author Contributions

YC, LL, and RW designed the experiments and interpreted the data. YC collected and analyzed the data and drafted the manuscript. LL, RW, and MW provided the critical revisions of the manuscript. All authors contributed to the article and approved the submitted version.

## Conflict of Interest

The authors declare that the research was conducted in the absence of any commercial or financial relationships that could be construed as a potential conflict of interest.

## Publisher’s Note

All claims expressed in this article are solely those of the authors and do not necessarily represent those of their affiliated organizations, or those of the publisher, the editors and the reviewers. Any product that may be evaluated in this article, or claim that may be made by its manufacturer, is not guaranteed or endorsed by the publisher.
